# Supplementary material for: Autapses enhance bursting and coincidence detection in neocortical pyramidal cells
Source: Nat Commun. 2018 Nov 20;9:4890. doi: 10.1038/s41467-018-07317-4 (PMC6244208; doi:10.1038/s41467-018-07317-4)
Supplement: Supplementary file 1 — Supplementary Information [file 41467_2018_7317_MOESM1_ESM.pdf]

Autapses enhance bursting and coincidence detection in neocortical pyramidal cells

Yin[1,2,#], Rui Zheng[1,2,#], Wei Ke[1,#], Quansheng He[1,#], Yi Zhang[1], Junlong Li[1], Bo Wang[1,2], Zhen Mi[1], Yue-sheng Long[4], Malte J. Rasch[1], Tianfu Li[3], Guoming Luan[3], Yousheng Shu[1,*]

Supplementary Information

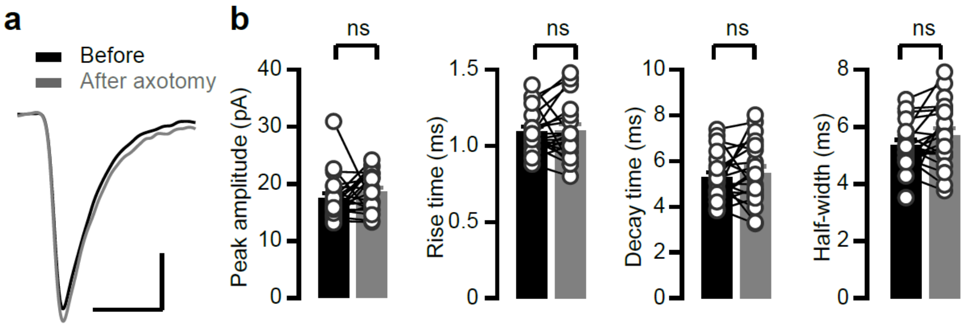

Supplementary Figure 1 Axotomy has no effect on the waveform of background spontaneous EPSCs. (**a**) Representative average traces of spontaneous EPSCs before (black) and after axotomy (gray). Scale bars: 10 ms/5 pA. (**b**) No significant changes in peak amplitude, rise time, decay time and half-width were detected after axotomy (*n* = 21 cells). Data are represented as mean ± SEM. ns, not significant, Wilcoxon signed-rank test for amplitude and paired Student's *t*-test for other comparisons.

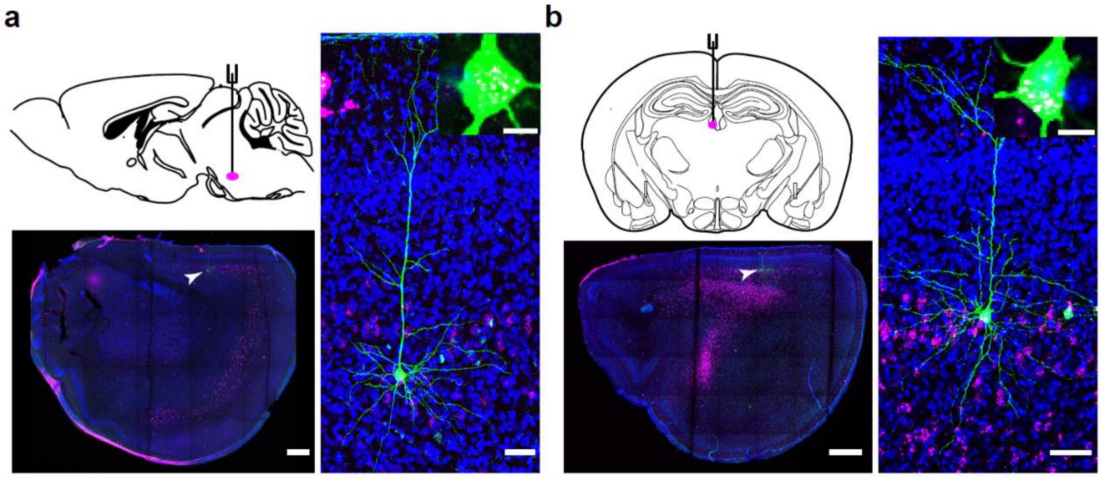

Supplementary Figure 2 Identification of PCs in PFC projecting subcortically to the pons and habenula. (**a**) Retrograde beads were injected to the pons (top left). Three days after injection, cortical cells in layer 5 were labeled by retrograde beads (magenta), indicating that they represented corticopontine projecting PCs (bottom left, scale bar: 500 μm). The arrowhead indicates a recorded cell that that was labeled by beads. Right, higher magnification of the recorded cell, which had been filled with biocytin during recording and stained green (scale bar: 50 μm). A single image of the cell body indicates that the cell contains beads (scale bar: 10 μm). Blue, DAPI staining. (**b**) Similar as in **a**, but with beads injection to the habenula.

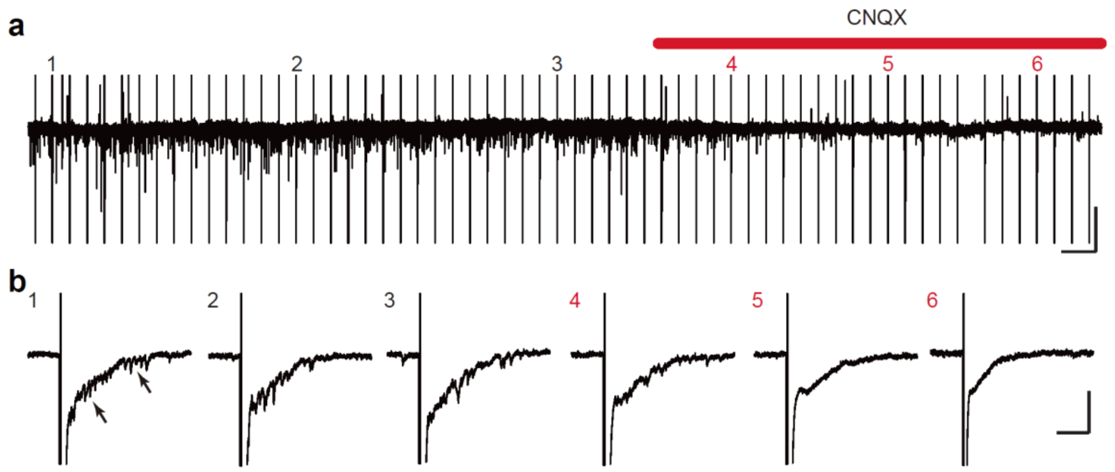

Supplementary Figure 3 Human layer-5 PC possesses autapses. (**a**) Example voltage-clamp recording from a layer-5 PC in human frontal cortex. The cell was stimulated with single voltage pulses (2 ms in duration, every 10 s) from the holding potential -70 mV to 50 mV. The bath solution contained 8 mM $SrCl_2$. Note that the background synaptic currents could be blocked by the application of 20 μM CNQX. Scale bars: 20 s/50 pA. (**b**) Six current responses evoked by voltage pulses are expanded. Note the presence and absence of autaptic events (arrows) before and after CNQX application. Scale bars: 100 ms/50 pA.

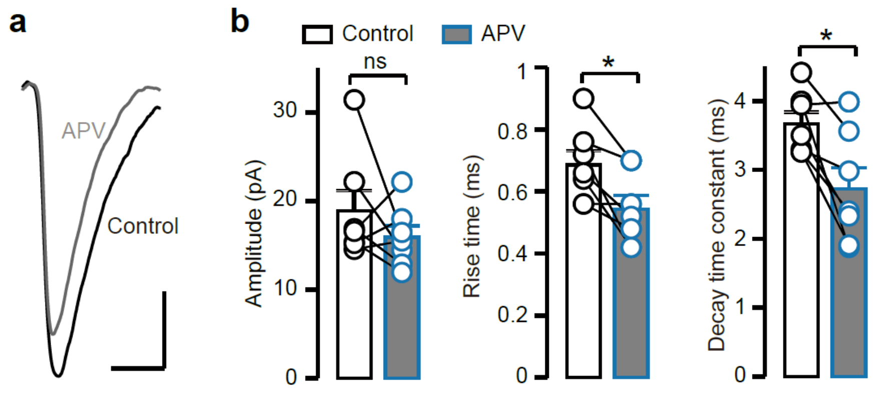

Supplementary Figure 4 Recurrent synaptic responses contain NMDA component. (**a**) Example desynchronized EPSCs before (black) and after the application of 50 μM APV (gray). Similar experiments as in **Fig. 5a** but with extracellular stimulation (see Methods) at a location nearby the recorded layer-5 PC in the absence of TTX. Scale bars: 5 ms/5 pA. (**b**) Changes of the peak amplitude, rise time and decay time constant of the evoked desynchronized EPSCs before and after the application of APV (paired Student's *t*-test). Data are represented as mean ± SEM. *, $P < 0.05$; ns, not significant.

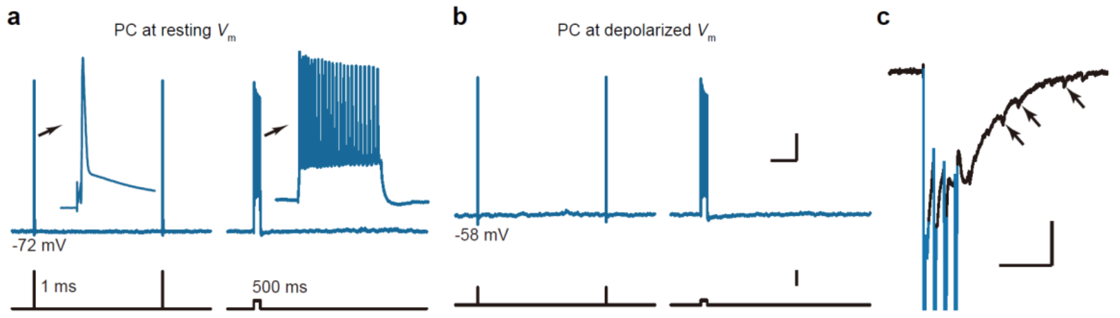

Supplementary Figure 5 Absence of persistent activity in autaptic PCs in slice preparation. (**a**) Current pulses (1 ms and 500 ms in duration) could not induce prolonged firing at a resting V_m of -72 mV. (**b**) No persistent activity could be observed even when the V_m was depolarized to a level near AP threshold. Scale bars: 2 s/20 mV for V_m and 1 nA for current. (**c**) The recorded PC could generate autaptic currents after bathing with Sr^{2+}-ACSF. Scale bars: 100 ms/50 pA.
